# The three lives of Pierre Boulanger

**DOI:** 10.1186/s12977-020-00518-0

**Published:** 2020-04-30

**Authors:** Nathalie Chazal, Hugues de Rocquigny, Philippe Roussel, Serge Bouaziz, Françoise Barré-Sinoussi, Jean-François Delfraissy, Jean-Luc Darlix

**Affiliations:** 1 IRIM, UMR 9004 CNRS-UM 1919, Route de Mende, 34293 Montpellier Cedex 5, France; 2 Morphogenèse et Antigénicité du VIH et des Virus des Hépatites, Hôpital Bretonneau, Inserm–U1259 MAVIVH, 10 Boulevard Tonnellé, BP 3223 37032 Tours Cedex 1, France; 3 Department of Biochemistry and Molecular Biology, Faculty of Medicine, Lille University, Lille, France; 4 Université Paris Descartes-UFR de Pharmacie, CiTcom CNRS UMR 8038, 4 Avenue de l’Observatoire, 75270 Paris Cedex 06, France; 5 Institut Pasteur, 75 Paris, France; 6 National Ethical Consultative Committee for Life Sciences and Health, 66 rue de Bellechasse, 75007 Paris, France; 7 Faculty of Pharmacy, CNRS UMR 7021, Strasbourg University, Illkirch, France


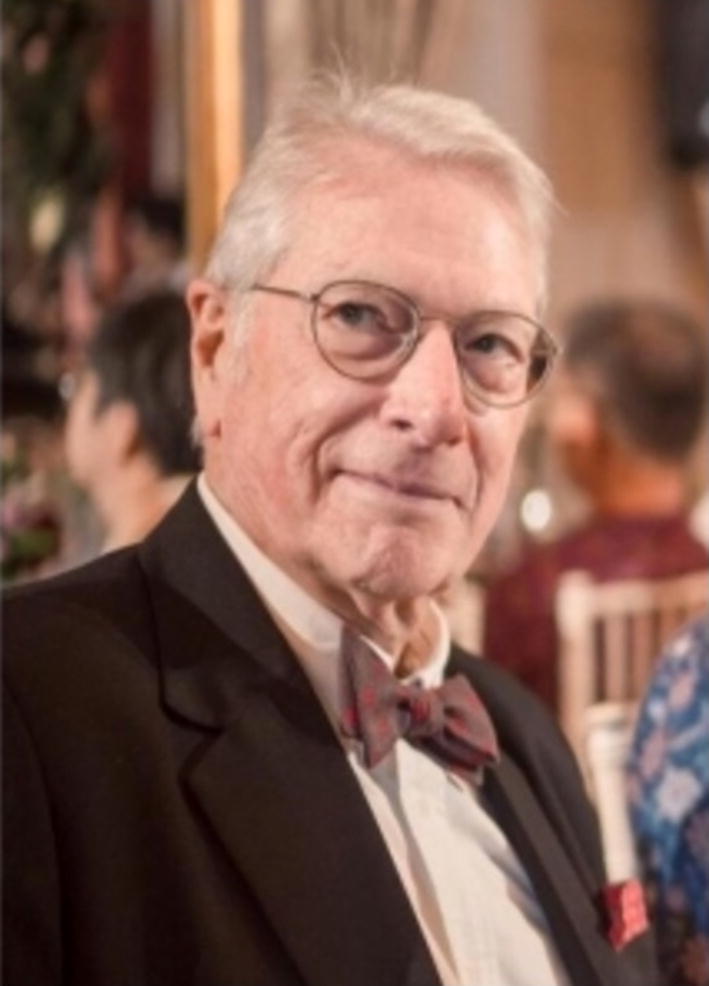
Pierre Boulanger, born on January 26, 1940, and died on March 30, 2020, was a renowned virologist with more than 30 years of experience in research and teaching in molecular virology and imaging, notably on Adenoviruses and HIV-1. After getting his medical degree at Lille Faculty of Medicine (France), Pierre Boulanger started his career in the Biochemistry Department being in charge of the INSERM “Molecular Virology” Laboratory #233, from 1978 to 1988. In 1988, Pierre moved to Montpellier, where he became Director of the Virology Department at the Montpellier Faculty of Medicine and Director of the CNRS UMR 5812 “Molecular virology” laboratory (UMR #5812). Pierre moved to Lyon in 1999 as Director of the Virology Department at Laennec Faculty of medicine at University Lyon 1 (1998–2006) and of the “Molecular Virology and Viral Pathogenesis” CNRS UMR5537 lab up to 2005. Professor Emeritus at Claude Bernard University, he joined the UMR754 “Viral infections and comparative pathology” laboratory.

During his career, Pierre’s research mainly focused on the structure–function relationships of viral proteins in virus assembly as well as on inhibitors targeting virus assembly. He identified and characterized cellular receptors of adenoviruses and developed effective capsid modification systems. He is the co-inventor of thirteen patents in the fields of adenoviral receptors, adeno-vectors, and baculoviruses. Pierre Boulanger was the co-author of more than 140 publications and reviews in international journals as well as book chapters on basic and medical virology and human gene therapy.

Pierre Boulanger also spent a lot of time and energy as president of two ANRS scientific committees (French National Agency for AIDS Research), and vice-president of the INSERM scientific council. He was a member of the VLM (Vaincre la Mucoviscidose) strategic committee, of the INRA scientific council, and the scientific committee of “GIANT,” a European consortium of laboratories dedicated to the treatment of prostate cancer using gene therapy.
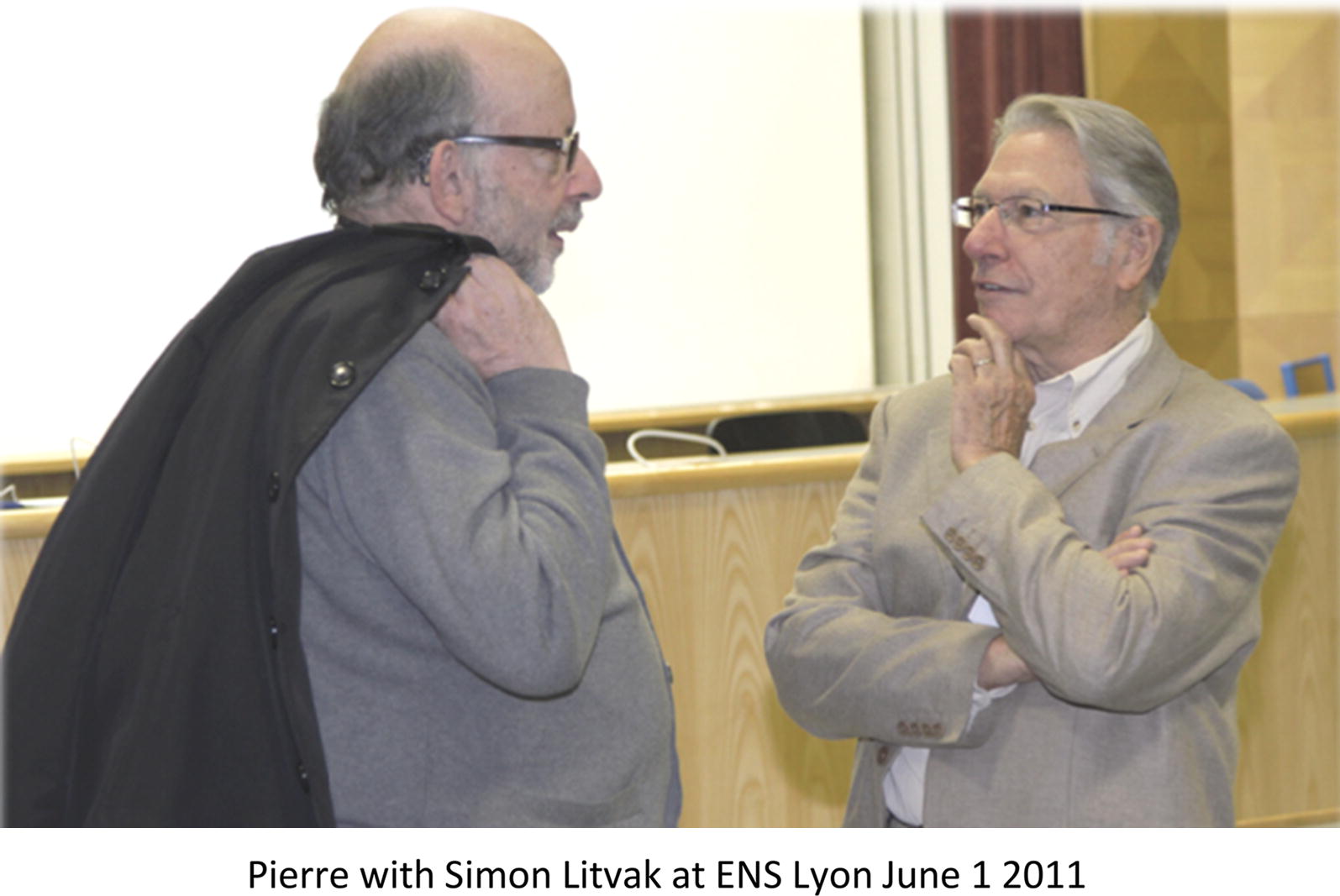



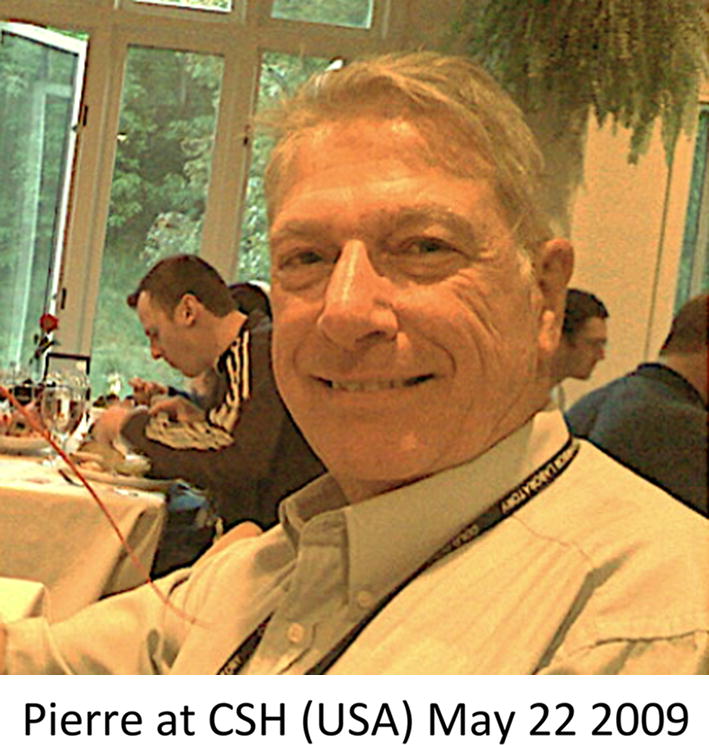


Pierre was indeed full of energy and enthusiasm to live three lives in Lille, Montpellier, and Lyon; Three lives for his family along with his duties as a medical doctor, as a post-doctoral investigator in three different virology labs and as a mentor and research director investigating adenovirus and HIV and at the same time developing viral vectors for gene transfer.

Pierre experienced three open lives as being a very open person, as a researcher sharing his latest data before publication with an open-access mentality and, at the same time, going straight to the points raised by competitors but never aggressive and always constructive in his comments.

Three secret lives as being a very humble person despite his very nice achievements, also giving us strong support without asking for a coauthorship and as a friend listening to personal problems and at the end telling us to be as positive as possible because life is lovely but short.

Pierre also experienced three lives in molecular virology, investigating the structure of Adenovirus particles and on HIV Gag assembly and at the same time at the forefront of the development of viral vectors for gene transfer and therapeutic purposes.

Regarding viruses, Pierre had a sceptical attitude concerning the general assumption that viruses are only poisonous agents. Such an assumption is indeed a global human issue, but are viruses causing diseases in most if not all instances or are viruses just the consequences of human overexploitation of all resources resulting in many negative impacts such as global warming, pollution, and reduction of biodiversity.

One of the critical questions he insisted is, “Are Viruses, friends or foes?”

Pierre was fascinated by the HIV Gag polyprotein precursor.

Since the years 1990, Pierre was pretty much interested in HIV virion formation, budding, and maturation. To that end, Nathalie joined his lab as a Ph.D. student and, together with Saw-See, set up an HIV- Gag recombinant baculovirus system for investigating particle formation in insect cells. Using this recombinant heterologous expression system, they soon discovered major Gag sites essential for Gag assembly and particle formation, notably the major homology region (MHR), the CA-NC spacer, and NC region. This pioneering work gave rise to critical publications and kick-started investigations on inhibitors targeting Gag assembly and maturation. Inhibitors were soon discovered such as Bevirimat and recently the EP39 molecule in excellent collaboration with Serge Bouaziz at the University of Paris Descartes; They discovered EP39 as being more water-soluble and more active than the original molecule and to inhibit assembly at high concentration while it was effective at low concentration on Gag processing. A European patent recently extended to the USA was recently filled out.

We will remember him as an affable, courteous, generous man, passionated by viruses and life, who loved discussing many issues about the virus world, especially with young students.

Our warmest thoughts are for his wife and Dear colleague Saw-See Hong, her lifelong scientific partner, and to his three children and grandchildren.

## Major publications

Boulanger, P. 1975. Adenovirus assembly : self-assembly of partially digested hexons. Journal of Virology, 16 : 1678-1682.

D’Halluin, J.C., Martin, G.R., Torpier, G. and Boulanger, P. 1978. Adenovirus type 2 assembly analyzed by reversible cross-linking of labile intermediates. Journal of Virology, 26 : 357-363.

D’Halluin, J.C., Milleville, M., Martin, G.R. and Boulanger, P. 1980. Morphogenesis of human adenovirus 2 studied with fiber- and fiber and penton base-defective temperature-sensitive mutants. Journal of Virology, 33 : 88-99.

Yoshinaga, S.K., Boulanger, P.A. and Berk, A.J. 1987. Resolution of human transcription factor TFIIIC into two functional components. Proceedings of the National Academy of Sciences, USA, 84: 3585-3589.

Hong, S.S. and Boulanger, P. 1993. Self-assembly-defective dominant mutants of HIV-1gag phenotypically expressed in baculovirus-infected cells. Journal of Virology, 67 : 2787-2798.

Chazal, N., Carrière, C., Gay, B. and Boulanger, P. 1994. Phenotypic characterization of insertion mutants of the human immunodeficiency virus type 1 Gag precursor expressed in recombinant baculovirus-infected cells. Journal of Virology, 68, 111-122.

Huvent, I., Hong, S.S., Fournier, C., Gay, B., Tournier, J., Carrière, C., Vigne, R., Spire, B & Boulanger, P. 1998. Interaction and co-encapsidation of human immunodeficiency virus type 1 Vif and Gag recombinant proteins. Journal of General Virology, 79, 1069-1081.** The front cover of this Journal issue reproduces one of the figures of our article.

Peytavi, R., Hong, S.S., Gay, B., Dupuy d’Angeac, A., Selig, L., Bénichou, S., Benarous, R., and Boulanger, P. 1999. HEED, the product of the human homolog of the murine eed gene, binds to the matrix protein of HIV-1. Journal of Biological Chemistry, 274, 1635-1645.

Gaden, F., Franqueville, L., Hong, S.S., Legrand, V., Figarella, C. and Boulanger, P. 2002. Mechanism of restriction of normal and CFTR-deficient human tracheal gland cells to Adenovirus (Ad) infection and Ad-mediated gene transfer. American Journal of Respiratory Cell and Molecular Biology, 27, 628-640.

Violot, S., Hong, S.S., Rakotobe, D., Petit, C., Gay, B., Moreau, K., Billaud, G., Priet, S., Schwartz, O., Sire, J., Mouscadet, J.-F. & Boulanger, P. 2003. The human Polycomb-group EED protein interacts with the integrase of human immunodeficiency virus type 1 (HIV-1). Journal of Virology, 77, 12507-12522.

Rakotobe, D., Tardy, J.-C., André, P., Hong, S.S., Darlix, J.-L., & Boulanger, P. 2007. Human Polycomb group EED protein negatively affects HIV-1 assembly and release. Retrovirology, 4 : 37.

Granio, O., Ashbourne Excoffon, K.J.D., Henning, P., Melin, P., Gonzalez, G., Karp, P.H., Habib, N., Lindholm, L., Becq, F., Boulanger, P., Zabner, J., & Hong, S.S. 2010. Adenovirus 5-fiber 35 chimeric vector mediates efficient apical correction of the cystic fibrosis transmembrane conductance regulator defect in cystic fibrosis primary airway epithelia. Human Gene Therapy, 21, 1-19.

Nangola, S., Urvoas, A., Valerio-Lepiniec, M., Khamaikawin, W., Sakkhachornphop, S., Hong, S.S., Boulanger, P., Minard, P., & Tayapiwatana, C. 2012. Antiviral activity of recombinant ankyrin targeted to the capsid domain of HIV-1 Gag polyprotein. Retrovirology, 9:17.

Dafonseca S, Coric P, Gay B, Hong SS, Bouaziz S, Boulanger P. The inhibition of assembly of HIV-1 virus-like particles by 3-O-(3′,3′-dimethylsuccinyl) betulinic acid (DSB) is counteracted by Vif and requires its Zinc-binding domain. Virol J. 2008 Dec 23;5:162.

DaFonseca S, Blommaert A, Coric P, Hong SS, Bouaziz S, Boulanger P. The 3-O-(3′,3′-dimethylsuccinyl) derivative of betulinic acid (DSB) inhibits the assembly of virus-like particles in HIV-1 Gag precursor expressing cells. Antivir Ther. 2007;12(8):1185-203.

